# First putative occurrence in the fossil record of choanoflagellates, the sister group of Metazoa

**DOI:** 10.1038/s41598-022-26972-8

**Published:** 2023-01-23

**Authors:** Carolina Fonseca, João Graciano Mendonça Filho, Matías Reolid, Luís V. Duarte, António Donizeti de Oliveira, Jaqueline Torres Souza, Carine Lézin

**Affiliations:** 1 Laboratório de Palinofácies e Fácies Orgânica (LAFO), Departamento de Geologia, Instituto de Geociências, Universidade Federal do Rio de Janeiro, Av. Athos da Silveira, 274, prédio do CCMN, sala J1020, Campus Ilha do Fundão, Cidade Universitária, Rio de Janeiro, RJ CEP 21.949-900 Brazil; 2 Universidade de Coimbra, MARE – Centro de Ciências do Mare do Ambiente, ARNET - Aquatic Research Network, Departamento de Ciências da Terra, Rua Sílvio Lima, 3030-790 Coimbra, Portugal; 3 Departamento de Geología and CEACTEMA, Universidad de Jaén, Campus Las Lagunillas sn, 23071 Jaén, Spain; 4 Université Toulouse III – Paul Sabatier, OMP, GET (Géosciences Environnement Toulouse), CNRS, IRD, 14 Avenue Édouard Belin, 31400 Toulouse, France

**Keywords:** Palaeontology, Petrology, Evolution

## Abstract

Choanoflagellates are microeukaryotes that inhabit freshwater and marine environments and have long been regarded as the closest living relatives of Metazoa. Knowledge on the evolution of choanoflagellates is key for the understanding of the ancestry of animals, and although molecular clock evidence suggests the appearance of choanoflagellates by late Neoproterozoic, no specimens of choanoflagellates are known to occur in the fossil record. Here the first putative occurrence of choanoflagellates in sediments from the Cretaceous (Cenomanian–Turonian) is described by means of several cutting-edge petrographic techniques, and a discussion of its paleoenvironmental significance is performed. Furthermore, their placement in the organic matter classification systems is argued, with a placement in the Zoomorph Subgroup (Palynomorph Group) of the dispersed organic matter classification system being proposed. Regarding the ICCP System 1994, incorporation of choanoflagellates is, at a first glance, straightforward within the liptinite group, but the definition of a new maceral may be necessary to accommodate the genetic origin of these organisms. While modern choanoflagellates may bring light to the cellular foundations of animal origins, this discovery may provide an older term of comparison to their extant specimens and provide guidelines for possible identification of these organic components in other locations and ages throughout the geological record.

## Introduction

Choanoflagellates are single-celled and colony-forming microeukaryotes, considered to be the closest living relatives of animals, and can be found in marine and freshwater environments. The interest in the evolutionary biology of choanoflagellates has spiked in the last decades, as they have the potential to reveal the genetic and cell biological foundations of animal cell differentiation^1–7^, due to their position as the sister-group to Metazoa in the eukaryotic supergroup Opisthokonta.

Choanoflagellate cells are characterized by bearing a single apical flagellum surrounded by a collar of microvilli, which together form a “collar complex”^8^. This structure is closely related with its feeding behavior, with the flagellar movement generating flows that attract bacteria onto the outer surface of the collar, where they are phagocytosed. This cell morphology is conserved in all choanoflagellate species and, within animals, is structurally and functionally preserved in the form of choanocytes, which constitute a group of specialized feeding cells that can be found in sponges. The structural and morphological resemblance between sponges and choanoflagellates choanocytes has been used since the nineteenth century as evidence of a close relationship between animals and these microeukaryotes^9–11^. Nevertheless, only modern molecular phylogenetic analyses provided conclusive evidence that sponges are members of the animal kingdom^12–14^, and that choanoflagellates are indeed the sister group of animals^1,5,14–17^. In addition, the last common ancestor of animals and choanoflagellates may resemble a modern choanoflagellate, as suggested by the phylogenetic relationship among choanoflagellates, sponges and eumetazoans^4,6,9,18^.

While choanoflagellates are vital players for the understanding of the ancestry of animals, and while molecular clock evidence suggests the appearance of choanoflagellates by late Neoproterozoic, their fossil record is virtually non-existent, with no known specimens being described in the literature^19–23^. Indeed, a multitude of studies in organic residues, isolated using highly sophisticated techniques, have been published throughout the years [e.g.,^24–33^]. Nevertheless, there is no known occurrence of an organic-walled organism with the same morphological characteristics as choanoflagellates.

With these premises, and based on the study of a Cretaceous sedimentary succession, the main objectives are: (i) to characterize the morphological features of choanoflagellates in a Cretaceous sedimentary succession using several cutting-edge petrographic techniques; (ii) to discuss the contribution of this microeukaryote to paleoenvironmental characterization; (iii) to include choanoflagellates in the dispersed organic matter classification system; and, (iv) to argue the position of this component in the ICCP (International Committee for Coal and Organic Petrology) System 1994.

## Stratigraphic and sedimentary setting

**Figure 1:**
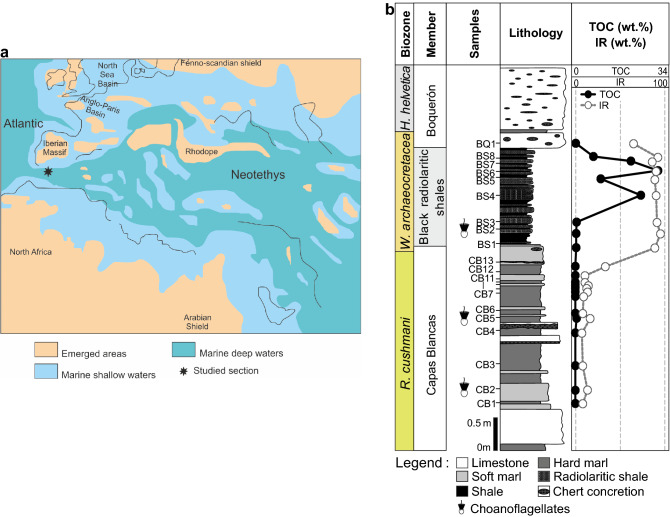
Baños de la Hedionda Section. (**a**) Paleogeographic map of the Cenomanian–Turonian with location of the studied section (modified from Reolid et al.^57^. (**b**) Stratigraphic logs of the Baños de la Hedionda Section. Highlight of sample location, and identification of samples with choanoflagellates in the *R. cushmani* and *W. archaeocretacea* biozones (CB2, CB5 and BS1). Total organic carbon (TOC) and insoluble residue (IR) values are plotted for each sample, with highest values being observed in the Black radiolaritic shales Member (average 15.78 wt.% and 89 wt.%, respectively).

The present study was performed in the Baños de la Hedionda section, located in the Betic Cordillera, Málaga Province (southern Spain; Fig. 1). This domain is divided into internal and external zones, with the last one comprising the Prebetic and Subbetic. These comprehend the record of epicontinental and epioceanic environments, respectively, starting from the early Jurassic^34,35^. The studied section is positioned in the W part of the Internal Subbetic, also called Penibetic, and represents the sedimentary record of a deep pelagic plateau located in the most distal part of the South Iberian Paleomargin^35^.

This Cenomanian–Turonian sedimentary succession comprises 6 m of the upper part of the Capas Blancas Formation (Fm.), represented here by the Capas Blancas Member (Mb.; 3.2 m of marls and marly-limestones with local chert nodules belonging to the *Rotalipora cushmani* and base of the *Whiteinella archaeocretacea* biozones), Black radiolaritic shales Mb. (1.45 m of thin laminated black clays and black radiolaritic cherts from the *W. archaeocretacea* Biozone) and Boquerón Mb. (1.3 m of white limestones with chert nodules and marls belonging to the top of the *W. archaeocretacea* and *Helvetoglobotruncana helvetica* biozones)^35^ (Fig. 1). The Cenomanian–Turonian boundary is located within the *W. archaeocretacea* Biozone^35^.

The kerogen present in the samples from the Baños de la Hedionda section was analysed using organic petrology in whole-rock polished blocks, then isolated and analysed using organic petrography, scanning electron microscopy and confocal laser scanning microscopy. Total organic carbon content and insoluble residue were also determined.

Total organic carbon content of the samples ranges between 0.01 and 31.48 wt.%, with the highest average values being recorded in the Black radiolaritic shales Mb. (15.78 wt.%), and the lowest in the Capas Blancas Mb. (Fig. 1). Samples from the Black radiolaritic shales Mb. show higher insoluble residue values (average 89 wt.%), while the lowest are observed in the Capas Blancas Mb. (average 12 wt.%; Fig. 1). The complete petrographic analysis of the kerogen allowed the identification, in several samples from the Capas Blancas and the base of the Black radiolaritic shales members, of one new organic component, choanoflagellates, until now never described in the worldwide fossil record.

## Choanoflagellates: the present is the key to the past

**Figure 2:**
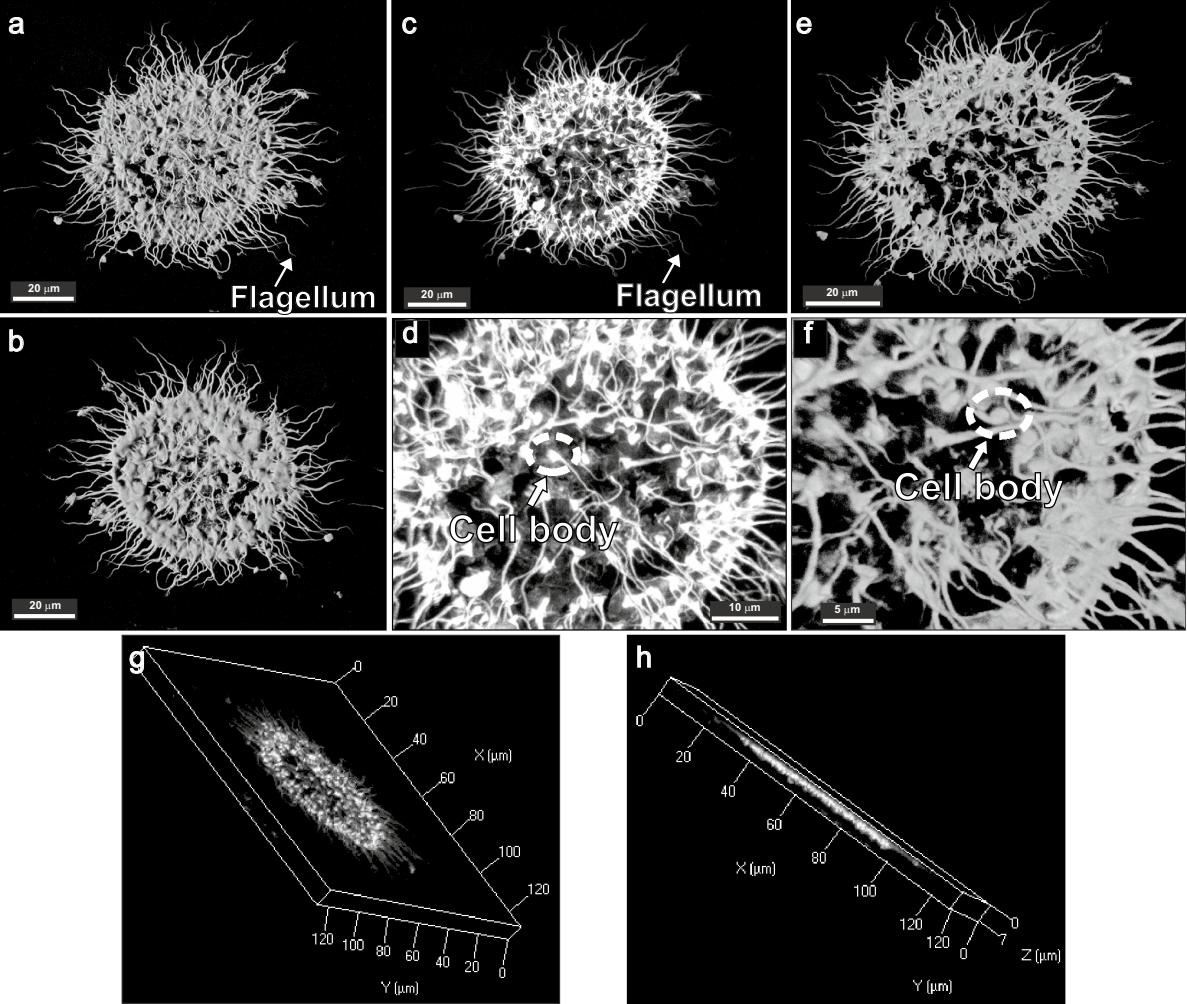
Confocal laser scanning microscopy photomicrographs of colonial choanoflagellate from the Capas Blancas Mb. (*R. cushmani* Biozone) at Baños de la Hedionda, with distinguishable cell body, flagellum, and the connecting tissue between each unicellular entity. (**a**) Front view with shadow filter. (**b**) Back view with shadow filter. (**c**) Front view with maximum filter. (**d**) Detailed zoom of figure c. (**e**) Front view with transparent filter. (**f**) Detailed zoom of figure e. (**g**–**h**) 3D model with X, Y and Z axis.

Choanoflagellates are single-celled and colony forming eukaryotes (3–10 μm) that lack a chloroplast belonging to the Superkingdom Eukaryota, Kingdom Protozoa, Phylum Choanozoa, Class Choanoflagellatea, and have long been considered as a transitional link between flagellated protists and sponges, or more precisely, as the closest living relatives of Metazoa^36–38^. They are apparently identical to the flagellated feeding cells of sponges (choanocytes), presenting a highly distinctive morphology. They are characterized by a unique spherical or ovoid cell topped by a fine collar (imperceptible by light microscopy) of actin-filled microvilli (“collar complex”) surrounding a single apical flagellum. Structural integrity of the colonies is ensured by a combination of intercellular bridges, extracellular matrix, and filopodia^8,39^. These morphological features, with the exception of the collar complex, were observed and identified in detail using confocal laser scanning microscopy to build a 3D model of a choanoflagellate specimen from the Baños de la Hedionda samples (Fig. 2). This model allowed for the observation of the front and back view of the choanoflagellate, allowing for an overall definition of the round structure of the colony (Fig. 2a,b,c,e). The use of the transparent and maximum filters allowed for the identification, in detail, of the singular apical flagellum and cell body of each singular member of the colony (Fig. 2c,d,e,f). The thinness of the specimen can also be appreciated (Fig. 2g,h). While a distinguishable cell body and the flagellum were identified, the connecting tissue between each unicellular entity is more difficult to pinpoint using this technique most likely since the distance between the slices (0.06 μm) during the scanning of the particle may not have enough resolution.

**Figure 3:**
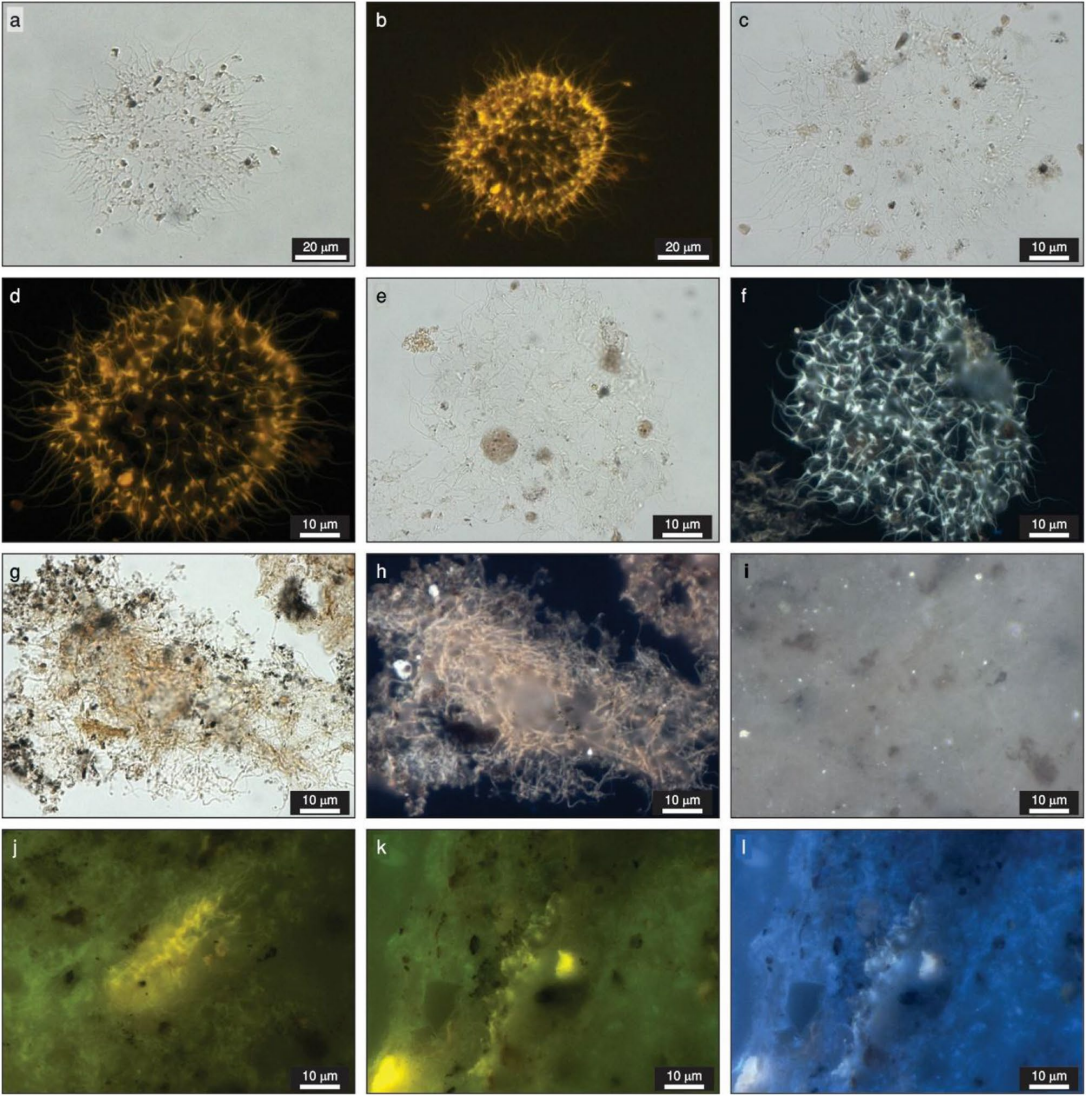
Photomicrographs of choanoflagellates from the Capas Blancas Mb. (*R. cushmani* Biozone) at Baños de la Hedionda. (**a**,**b**,**c**,**d**), Choanoflagellate with high preservation state (palynofacies slide). (**e**,**f**,**g**,**h**), Amorphous choanoflagellate (palynofacies slide). (**i**,**j**,**k**,**l**), Choanoflagellate (whole-rock). Transmitted white light: (**a**,**c**,**e**,**g)**. Reflected white light: (**i**). Fluorescence mode: (**b**,**d**,**f**,**h**,**j**,**k**,**l**).

**Figure 4:**
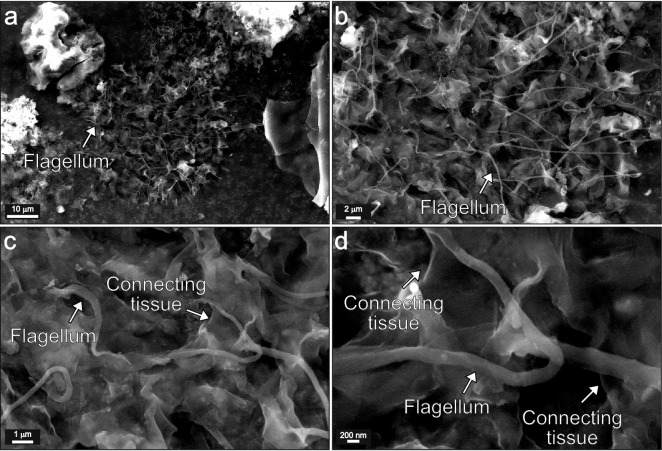
Scanning electron microscopy analysis. (**a**, **b**, **c**, **d**) Photomicrographs of colonial choanoflagellate from the Capas Blancas Mb. (*R. cushmani* Biozone) at Baños de la Hedionda, highlighting the flagellum and the connecting tissue that joins the individual cells of the colonial choanoflagellate.

In the isolated kerogen palynofacies slides, choanoflagellates present the same morphological characteristics as observed in confocal laser scanning (Fig. 2), exhibiting a colonial aspect, and the possible identification of the cell body and the single apical flagellum (Fig. 3). Regarding its morphographic features, the specimens with highest degree of preservation are almost transparent in transmitted white light, presenting a dark yellow fluorescence under blue incident light (Fig. 3a,b,c,d). The particles with higher degree of amorphization (microbiological reworking by anaerobic bacteria) display a light brownish color in transmitted white light, and a dark yellow fluorescence under blue incident light (Fig. 3e,g,h). In whole-rock polished-blocks, choanoflagellates are transparent under reflected white light and exhibit a dark yellow fluorescence (Fig. 3i,j,k,l). Scanning electron microscopy allowed for the observation, in detail, of the connecting tissue that joins the individual cells to form the colonial choanoflagellate identified in the studied samples (Fig. 4).

In the choanoflagellate observed in the studied samples, the typical collar complex is absent, which could be considered problematic to their identification and classification. Although this morphological feature is easily recognized in present day choanoflagellates, fossilization processes need to be considered when analysing the studied specimens. It is well established that the microvilli of the collar are primarily composed of actin microfilaments^40,41^. Thus, the preservation potential of the microvilli is extremely low to nonexistent^42^. The sedimentary organic matter is derived from live organic matter and from products of its metabolism. Living tissues are composed of an assembly of thermodynamically unstable biomolecules. When such molecules are secreted or excreted by the living organisms, or even after their death, they tend to lose their integrity and can be transformed into simple stable components (e.g., CO_2_, CH_4_, NH_3_, H_2_S, H_2_O, etc.). This degradation process can be accomplished by the physicochemical processes (oxidation, photolysis, thermolysis, hydrolysis, etc.)^42^. The destructive action will depend on the specific resistance of each of the different biochemical entities, which will determine the accumulation of organic matter^43^. The components more susceptible to decomposition are proteins, which are broken down into water-soluble monomers (amino acids), due to the action of enzymes. These are followed by carbohydrates, which also are broken down into simple sugars by enzymatic action, while lipids and lignin are the most resistant components^42^. Taking this into account, it is fair to assume that the protein-rich collar of microvilli was not preserved, while other parts of the choanoflagellate, richer in lipids, were fossilized. The presence of a spherical or ovoid cell with a single apical flagellum in each unicellular entity allows the identification of these colonial organisms as choanoflagellates and distinguishes them from other organic-walled microfossils. The individual palynological colonial components that are usually encountered in geological palynological preparations encompass *Botryococcus*, *Pediastrum*, *Scenedesmus* and *Gloeocapsomorpha*^43,44^. The morphological characteristics of these components are completely different from the ones exhibited by the organic-walled microfossil identified as choanoflagellate. Furthermore, green algae from the order Chlamydomonadales (genus *Gonium* and *Volvox*) and golden algae from the class Chrysophyceae were also considered, but colonies are either not spherical or each individual entity displays two flagella^45,46^. Thus, this seems to be the first putative occurrence of Choanoflagellates in the fossil record.

## The paleoenvironmental significance of choanoflagellates

Present day choanoflagellates are abundant and globally distributed in marine and freshwater environments, inhabiting both pelagic and benthic environments (being recovered from as deep as 300 m^8,47–49^. They are one the most important bacteria-consuming groups bonding otherwise unreachable forms of carbon to higher trophic levels, and thus having a profound impact on marine microbial food webs and the global carbon cycle^36,38,50,51^. At Baños de la Hedionda, the Cenomanian–Turonian choanoflagellates occur together with an extremely diverse palynological association. The palynological association of the Capas Blancas Mb. is characterized by marine phytoplankton-derived amorphous organic matter (originated by microbiological reworking of marine phytoplankton by heterotrophic bacteria) and freshwater microplankton, with some specimens of marine microplankton (dinoflagellate cysts), sporomorphs, zoomorphs and phytoclasts being also identified. The palynological association of the Black radiolaritic shales Mb. displays an increase in the degree of amorphization of all the components, translating an overall transgressive trend^43^. In this member, the amorphous organic matter is dominant (with both marine phytoplankton and bacterial origin), with some particles of freshwater microplankton (*Botryococcus* sp.), marine microplankton (dinoflagellate cysts), sporomorphs and phytoclasts being also present. Although the association changes and evolves throughout the section, the mixture of freshwater and marine components is constant, which hinders the identification of the paleoenvironmental affinity of the choanoflagellates. At the base of the section, the freshwater components and the choanoflagellates show a low amorphization state, which may indicate some transport into the system as some marine phytoplankton-derived amorphous organic matter is present. At the base of the Black radiolaritic shales Mb. the choanoflagellates begin to present a higher amorphization state (oxygen deficiency allowed for the more effective amorphization processes by anaerobic microbiological reworking to occur), which is congruent with the development of reducing conditions (possibly euxinia) during this time^35^. The similar amorphization degree of choanoflagellates and freshwater microplankton may indicate a freshwater affinity of the choanoflagellates identified in this section, nevertheless, the discovery and examination of these components in other sections with different age, paleogeographical and paleoenvironmental contexts is key for a more reliable definition of the paleoenvironmental affinity of these components throughout the geological record.

## Choanoflagellates in the organic matter classification systems

Protozoa is undoubtedly a paraphyletic taxon, i.e., Animalia, Fungi, Plantae, and Chromista evolved from it^37,38^. Some protists, such as the choanoflagellates, are more closely related to animals than to other protists. Considering the dispersed organic matter classification system^43^, and its most recent update^44^, choanoflagellates should belong to the Palynomorph Group as, by definition, it includes all organic-walled microfossils remaining after acid maceration. In terms of subgroups, due to its close relationship with animals, a case may be made to include them, even if temporarily, in the Zoomorph Subgroup as it encompasses discrete individual animal-derived particles, whether whole or damaged^43^. Due to their presence in both marine and freshwater environments, another possibility is to create a new subgroup, such as the “Mixed Aquatic Palynomorph Subgroup” or the “Others Subgroup”, to accommodate these new components. The “Mixed Aquatic Palynomorph Subgroup” would include both freshwater and marine organic-walled aquatic constituents. The “Others Subgroup” may comprise aquatic organic-walled components (both freshwater and marine) that are neither animal-derived or photosynthetic, and others.

Following the ICCP System 1994 for maceral group classification^52^, according to the morphographic features and optical properties (reflectance and fluorescence) of choanoflagellates, these components should belong to the liptinite group. By definition, “liptinite is a group of macerals derived from non humifiable plant matter, relatively hydrogen rich remains such as sporopollenin, resins, waxes and fats and comprise the macerals of the lowest reflectance at a given rank among the other macerals”^52^. Although the taxonomy of choanoflagellates may be a constrain, this group is characterized by organic components that display a lower reflectance than vitrinite at low and medium rank and show autofluorescence when illuminated with ultra-violet and blue lights^52^, which is aligned with the optical properties of choanoflagellates.

The liptinite group is composed by nine macerals, from which only one, alginite, seems to encompass the optic and morphographic features of choanoflagellates. At first sight, choanoflagellates seem to present a filamentous, almost lamellae, morphology with limited thickness and lateral extent which may indicate that lamalginite (a subdivision of alginite) must be the better choice^52^ (Fig. 3k,l). Still, a more careful analysis of the individual organic particles allows the recognition of a punctuated or dotted structure, representing the individual members of the colony (Fig. 3j). Ultimately, choanoflagellates are not a planktonic or benthic algae, so it does not fit the definition of the organic components within this maceral^52^. This entails that the discussion of the classification of these new organic components within the ICCP System 1994 is still open, as none of the macerals currently established fits its origin and optical and morphographic features.

While the study of modern choanoflagellates holds the promise of illuminating the cellular foundations of animal origins^8^, this discovery may bring new light into the reconstruction of the development and evolution of choanoflagellates, whose first specimens evolved over 600 million years ago. This older term of comparison to the present day living choanoflagellates provides the guidelines for possible identification of these organic components in other locations and ages throughout the geological record.

## Methods

### Sample collection and specimen identification

A set of 22 samples was collected from the Baños de la Hedionda section, belonging to the Cenomanian—Turonian Capas Blancas Fm.: 13 from the Capas Blancas Mb., 8 from the Black radiolaritic shales Mb., and 1 from the Boquerón Mb. The studied samples did not show evidence of weathering.

### Total organic carbon and insoluble residue

Total organic carbon (TOC) content was determined (after acidification for removal of carbonates) using a LECO SC 144 analyzer and following standard procedures^53^. Insoluble residue represents the fraction of the sample not eliminated by acidification (presuming a total removal of the carbonates).

### Organic petrology

Optical petrography was performed on whole-rock polished blocks prepared according to ISO 7404-2^54^, utilizing a Zeiss Axioskop 2-plus microscope. Maceral identification was based on optical properties and morphology defined by ICCP System 1994^52^.

### Kerogen isolation and organic petrography

Standard non-oxidative procedures were used for isolation of the kerogen from the rock matrix^43,55^. Around 20 to 40 g of sediment were crushed (2 mm size) and underwent an acid treatment for removal of the rock matrix (carbonates—HCl 37% for 18 h; silicates—HF 40% for 24 h; neoformed fluorides—HCl 37% for 3 h). Then, kerogen was concentrated using a heavy liquid (ZnCl_2_, density = 1.9 to 2 g/cm^3^). Drops of HCl (10%) were added to the organic fraction followed by neutralization using distilled water. The material was centrifuged (3 min at 1500 rpm), and the excess water discarded. After decantation, strew slides (palynofacies slides) were prepared using the isolated kerogen (sieved at 10 μm). Petrographic analysis for organic particles identification was performed using transmitted white and incident blue (fluorescence mode) lights, following the dispersed organic matter classification^43,44^. A high number of choanoflagellate specimens were identified in the studied samples. The richest sample (sample CB2) underwent palynofacies analysis (qualitative and quantitative examination of the kerogen, counting of 300 to 500 particles)^43,44^, and choanoflagellates represent 15.5% of the palynological association of that sample. In other samples, choanoflagellates appear in smaller numbers and different preservation stages, which makes it difficult to quantify.

### Confocal laser scanning microscopy

For confocal laser scanning microscopy (CLSM) analysis, samples underwent panning to increase palynomorph recovery and then were sieved using nylon membranes of 40 μm and 20 μm-mesh^56^. CLSM images were acquired on a CLSM Zeiss LSM 700 confocal system equipped with a Zeiss Zen 2011 (Black edition) software. A 488 nm diode source, high sensitivity PMT (photomultiplier tube) detector with spectral increment of 1 nm, 100 × oil objective, distance between the slices of 0.06 μm, resolution of 2048 by 2048 pixels, 8-bit (bit-depth) were used.

### Scanning electron microscopy

For *s*canning electron microscopy analysis, a drop of the isolated kerogen was placed on a carbon ribbon, on an aluminium stub, and let dry for 24 h. Observations were performed using a Zeiss EVO M10 Scanning Electron Microscope, with 14.45 kV voltage (EHT) and working distance (WD) of 13.0 mm under vacuum.

## Data Availability

All data generated or analyzed during this study are included in this published article. All materials (rock samples and slides) are housed in the collections of the Palynofacies and Organic Facies Laboratory of the Federal University of Rio de Janeiro, Rio de Janeiro, Brazil.
